# Effect of Anemia on Physical Function and Physical Activity in CKD: The National Health and Nutrition Examination Survey, 1999–2016

**DOI:** 10.34067/KID.0000000000000218

**Published:** 2023-09-28

**Authors:** Youssef M.K. Farag, Elena Blasco-Colmenares, Di Zhao, Myrlene Sanon, Eliseo Guallar, Fredric O. Finkelstein

**Affiliations:** 1 Akebia Therapeutics, Inc., Cambridge, Massachusetts; 2 Department of Epidemiology, Johns Hopkins University School of Public Health, Baltimore, Maryland; 3 Department of Medicine, Welch Center for Prevention, Epidemiology, and Clinical Research, Johns Hopkins University School of Medicine, Baltimore, Maryland; 4 Otsuka Pharmaceutical Development and Commercialization, Inc., Princeton, New Jersey; 5 Yale University School of Medicine, New Haven, Connecticut

**Keywords:** anemia, CKD, National Health and Nutrition Examination Survey (NHANES), physical activity, physical functioning

## Abstract

**Key Points:**

- In a large sample representative of the US adult noninstitutionalized population, among participants with CKD stages 3–5, anemia was associated with a significantly lower level of physical activity.
- The presence of CKD and anemia showed a positive interaction on physical functioning outcomes. Among participants with CKD, physical functioning was worse in patients with anemia compared with those without anemia.

**Background:**

CKD is a major public health problem worldwide. Anemia, a frequent and treatable complication of CKD, is associated with decreased physical functioning and physical activity. The objective of this study was to evaluate the joint association of CKD and anemia with physical functioning and physical activity in a representative sample of the US population.

**Methods:**

Cross-sectional study using the National Health and Nutrition Examination Survey (NHANES) 1999–2016 for physical functioning outcomes (*N*=33,300) and NHANES 2007–2016 for physical activity (*N*=22,933). The NHANES physical functioning questionnaire included 19 items. The NHANES physical activity questionnaire captured work-related, leisure-time, and sedentary activities. Higher physical functioning scores represent worse function. CKD was classified using Kidney Disease Outcomes Quality Initiative 2002 criteria, and anemia was defined using the World Health Organization criteria.

**Results:**

The adjusted mean differences (95% confidence interval) in overall physical functioning score comparing participants with anemia with those without anemia among participants with no CKD, CKD stages 1–2, and stages 3–5 were 0.5 (−0.1 to 1.0), 1.5 (0.2 to 2.8), and 3.6 (2.0 to 5.2). Anemia and CKD showed a supra-additive interaction for all physical functioning outcomes among participants in CKD stages 3–5. The prevalence of high physical activity was also lower in participants with anemia compared with those without anemia among participants in CKD stages 3–5 (adjusted prevalence ratio, 0.74; 95% confidence interval, 0.54 to 1.01).

**Conclusions:**

CKD and anemia were associated with impairments in physical functioning and reduced physical activity. For physical functioning outcomes, the combined presence of CKD and of anemia showed a stronger effect than what was expected from their independent effects.

## Graphical abstract

**Figure:**
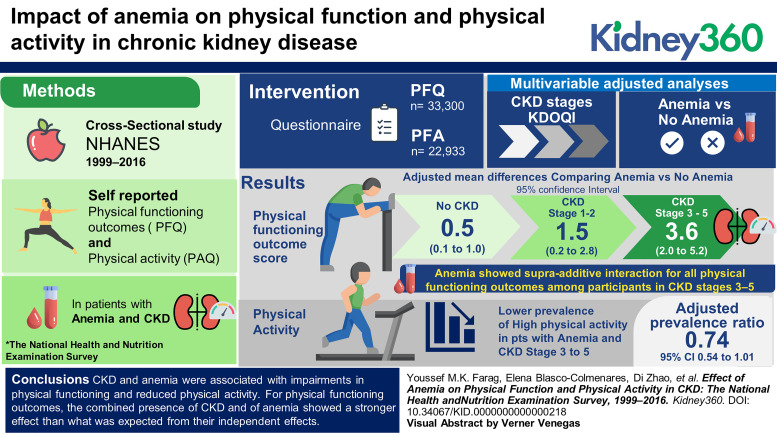

## Introduction

CKD is a major public health problem worldwide.^1^ In the United States, 30 million adults have CKD, and 124,500 new cases of kidney failure were reported in 2017.^2^ For the same year, the total Medicare expenditure in kidney disease was almost $120 billion.^2^ Furthermore, CKD is associated with increased risk of cardiovascular disease,^3,4^ mortality,^5^ physical disability, and impaired quality of life.^6^

Anemia is a frequent complication of CKD. The prevalence of anemia increases with CKD stage, from 8.4% in stage 1 to 53.4% in stage 5.^7^ Anemia is associated with decreased quality of life,^8^ functional capacity,^8^ and increased risk of cardiovascular events and mortality in advanced CKD stages.^9^ Anemia is associated with a decrease in physical functioning and physical activity in advanced CKD stages and in patients on dialysis, but the implications of anemia on physical functioning and physical activity across the whole spectrum of CKD have not been characterized. Physical functioning allowing for independent living is a major health concern in patients with different CKD stages,^10,11^ while anemia is a potentially treatable complication of CKD.^12,13^ Clinical trials that target increasing hemoglobin levels in patients with CKD showed conflicting results, with trials suggesting hemoglobin normalization by medications improved^14,15^ or did not affect physical function.^16–18^ As a consequence, the evaluation of the association of anemia with physical function and physical activity in patients with CKD is a clinically relevant concern.

In this study, we evaluated the association of CKD and anemia with self-reported physical function and self-reported physical activity in a representative sample of the US population. We hypothesized that more advanced CKD stages and the presence of anemia would be independently associated with decreased physical function and physical activity and that the combined presence of CKD and anemia would have a stronger association than each variable individually.

## Methods

### Study Sample

The National Health and Nutrition Examination Survey (NHANES) is a nationally representative survey designed to assess the health and nutritional status of the noninstitutionalized US population. NHANES examines a nationally representative sample of approximately 5000 persons each year. In NHANES, data on physical functioning were collected from 1999 to 2016 and physical activity from 2007 to 2016. For this analysis, we selected participants aged 20 years or older and excluded participants who did not participate in the mobile examination center (MEC) examination, were pregnant, had a history of cancer, had a positive HIV antibody test, received dialysis within the past 12 months, or had missing data on serum creatinine, urinary albumin-creatinine ratio, hemoglobin, or in the outcome or adjustment variables.

### Data Collection

NHANES included a household interview and an MEC examination. Sociodemographic information including age, sex, race, ethnicity, marital status, education, income, insurance, employments status, smoking, alcohol consumption, and previous medical conditions were collected using standardized questionnaires. Alcohol intake was categorized as <1, 1 to <3, or ≥3 drinks per week. Smoking was categorized as never, former, and current. Weight and height were measured, and body mass index was calculated as weight in kilograms divided by the square of height in meters. Hypertension was defined as a blood pressure >140/90 mm Hg or self-reported use of antihypertensive medication.

Blood samples for biochemical analyses were obtained during the MEC examination. Anemia status was defined using the World Health Organization criteria as hemoglobin <12 g/dl in women and <13 g/dl in men.^19^ Because we analyzed a general population sample, the number of participants with severe anemia was small and did not allow for separate subgroup analyses. eGFR was calculated using the CKD Epidemiology Collaboration formula.^20^ Albuminuria was defined as a urinary albumin-creatinine ratio ≥30 mg/g.^21^ CKD was classified using Kidney Disease Outcomes Quality Initiative 2002 criteria as CKD stage 1 if albuminuria was present and eGFR was ≥90 ml/min per 1.73 m^2^; CKD stage 2 if albuminuria was present and eGFR was 60–89 ml/min per 1.73 m^2^; CKD stage 3 if eGFR was 30–59 ml/min per 1.73 m^2^; CKD stage 4 if eGFR was 15–29 ml/min per 1.73 m^2^; and CKD stage 5 if eGFR was <15 ml/min per 1.73 m^2^.^22^

Hypercholesterolemia was defined as a total cholesterol ≥200 mg/dl,^23^ a self-reported doctor's diagnosis of hypercholesterolemia, or use of lipid-lowering medication. Diabetes was defined as a fasting serum glucose ≥126 mg/dl,^24^ a nonfasting serum glucose ≥200 mg/dl,^24^ a doctor's diagnosis of diabetes, or use of medication to lower blood sugar.

### Physical Functioning

Physical functioning questionnaire was collected in NHANES 1999–2006. We used these data in NHANES to evaluate functional limitations caused by long-term physical, mental, and/or emotional problems or illness. Physical functioning questionnaire questions were phrased to assess the level of difficulty in performing different tasks without using any special equipment. Functional limitations were assessed in five domains: lower extremity mobility (three items: walking for a quarter mile; walking ten steps; and stooping, crouching or kneeling), general physical activity (six items: lifting or carrying, standing up from armless chair, standing for long periods, sitting for long periods, reaching up over head, and grasping/holding small objects), activities of daily living (ADLs; four items: walking between rooms in the same floor, getting in and out of bed, using fork, knife, drinking from cup, and dressing yourself), instrumental ADL (IADLs; three items: managing money, house chores, and preparing meals), and leisure and social activities (three items: going out to movies/events, attending social events, and leisure activity at home). For each individual item, the response options were no difficulty, some difficulty, much difficulty, or unable to do the activity. These responses were scored from 0 to 3, and the scores of each item were added to obtain dominion scores and an overall physical function score. We then rescaled all physical functioning scores (dividing the original score by the maximum possible score and multiplied by 100) so that each score ranged from 0 to 100, with higher scores reflecting worse function.

### Physical Activity and Physical Fitness

Global physical activity questionnaire was collected in NHANES 2007–2016. Physical activity was captured in the NHANES which includes questions related to work, leisure time, and sedentary activities. Each activity was then assigned metabolic equivalent of task (MET) values (vigorous work-related activity, vigorous leisure-time physical activity, eight METs; moderate work-related activity, walking or bicycling for transportation, moderate leisure-time physical activity, four METs). An MET is a measure of the ratio of the rate of energy expenditure relative to the mass of that person while performing some specific physical activity compared with a reference, set by convention at 3.5 ml of oxygen per kilogram per minute, which is roughly equivalent to the energy expended when sitting quietly (1 MET=1 kcal/kg×hour).^25^ We calculated total MET-minutes by summing the MET-minutes for each activity and obtained a weekly MET-minute score for each participant. We categorized physical activity as insufficient (some regular activity, but not reaching the minimum standard of guidelines), moderate (500–1000 MET-minutes per week), and high (>1000 MET-minutes per week).

### Statistical Analysis

For continuous outcomes, we used linear regression to estimate mean differences in outcome variables across categories of CKD and anemia. For dichotomous and categorical outcomes, we used logistic and polychotomous logistic regression models, respectively. For dichotomous and categorical outcomes, we estimated marginally adjusted proportions and use them to compute prevalence ratios of these outcomes across categories of CKD and anemia.

For all outcomes, we fitted three models with increasing levels of adjustment. Model 1 adjusted for age, sex, race/ethnicity, and survey cycle. Model 2 further adjusted for marital status, education level, household income, health insurance and employment status, smoking status, alcohol use, and comorbidities (coronary heart disease, angina, myocardial infarction, stroke, chronic heart failure, chronic obstructive pulmonary disease, and arthritis). Model 3 further adjusted for diabetes, hypertension, and hyperlipidemia.

All statistical analyses were conducted using appropriate sample weights and primary sampling units to account for the complex sample design in NHANES. Analyses were conducted using survey (svy) commands in Stata 16 (StataCorp, College Station, TX).

## Results

### Physical Functioning

**Figure 1.**
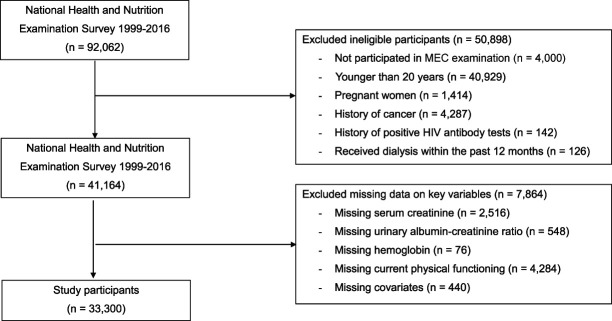
**Flowchart of study participants in the physical functioning component, National Health and Nutrition Survey 1999–2016.** MEC, mobile examination center.

For the physical functioning analyses (NHANES 1999–2016), 33,300 participants were eligible and had complete data (Figure 1). The mean (SEM) age was 46.7 (0.2) years; 49.0%, 67.7%, and 11.1% were male, non-Hispanic White, and non-Hispanic Black, respectively (Table 1). The proportion of participants who were married, had ≥college degree, had ≥$75,000 of annual household income, had health insurance, and were employed were 56.2%, 27.3%, 34.2%, 81.1%, and 64.7%, respectively. The proportion of current smokers and of participants who had ≥3 drinks per week were 21.9% and 18.8%, respectively. Diabetes, hypertension, and hyperlipidemia were present in 12.6%, 36.6%, and 67.8% of study participants, respectively. The prevalence of comorbidities increased with declining renal function and with the presence of anemia. The mean hemoglobin, eGFR, and albumin-creatinine levels in participants without and with anemia by increasing CKD category are shown in Table 1.

**Table 1.** Characteristics of study participants with physical functioning information, National Health and Nutrition Survey 1999–2016

Variable	Overall	No CKD		CKD Stages 1–2		CKD Stages 3–5	
		No Anemia	Anemia	No Anemia	Anemia	No Anemia	Anemia
*N* sample	33,300	25,601	1868	2776	374	2032	649
*N* population, ×10^6^	151.5	124.6	6.4	10.4	1.0	7.5	1.7
Age, yr	46.7 (0.2)	44.5 (0.2)	46.5 (0.5)	51.4 (0.4)	53.4 (1.0)	70.1 (0.3)	72.3 (0.4)
Sex, % male	49.0 (0.3)	51.5 (0.3)	23.0 (1.3)	44.8 (1.3)	33.1 (3.2)	39.3 (1.3)	42.5 (2.1)
**Race, %**							
Non-Hispanic White	67.7 (1.3)	69.0 (1.2)	40.6 (2.4)	61.7 (1.9)	33.8 (3.6)	82.3 (1.2)	63.9 (2.4)
Non-Hispanic Black	11.1 (0.6)	9.6 (0.6)	33.5 (2.1)	12.5 (1.0)	39.7 (3.2)	7.6 (0.7)	21.5 (1.8)
Mexican American	8.6 (0.7)	8.8 (0.7)	9.9 (1.1)	11.0 (1.0)	11.0 (2.1)	2.8 (0.4)	5.1 (0.9)
Other Hispanic	5.5 (0.5)	5.5 (0.5)	7.0 (0.8)	6.3 (0.7)	9.6 (1.7)	3.1 (0.5)	3.4 (0.8)
Other	7.1 (0.4)	7.1 (0.4)	9.1 (1.0)	8.5 (0.7)	5.9 (1.1)	4.2 (0.6)	6.0 (1.1)
Married, %	56.2 (0.6)	56.9 (0.6)	52.9 (1.7)	51.5 (1.4)	48.5 (3.1)	55.2 (1.5)	47.5 (2.5)
**Education, %**							
<9th grade	6.5 (0.3)	5.7 (0.3)	7.3 (0.6)	11.1 (0.7)	16.7 (2.4)	10.2 (0.8)	15.6 (1.6)
9th–11th grade	11.6 (0.4)	11.0 (0.4)	13.6 (1.0)	14.3 (0.7)	16.9 (2.5)	14.5 (1.0)	17.5 (1.9)
High school or GED	23.5 (0.4)	23.2 (0.5)	22.1 (1.2)	25.2 (1.2)	21.7 (2.8)	27.1 (1.3)	31.0 (2.1)
Some college or AA	31.0 (0.4)	31.3 (0.5)	32.6 (1.4)	30.4 (1.3)	26.7 (2.9)	26.1 (1.2)	24.9 (2.2)
≥College	27.3 (0.8)	28.8 (0.9)	24.4 (1.6)	19.0 (1.4)	18.0 (2.9)	22.0 (1.5)	11.0 (1.6)
**Household income, %**							
<$20k	16.1 (0.5)	14.3 (0.5)	20.6 (1.4)	23.8 (1.2)	31.3 (3.0)	25.7 (1.3)	33.2 (2.4)
$20k to <$35k	17.3 (0.4)	16.4 (0.4)	20.2 (1.3)	21.2 (1.0)	23.2 (3.1)	21.7 (1.0)	26.6 (2.4)
$35k to <$75k	32.4 (0.6)	33.0 (0.7)	29.9 (1.3)	29.6 (1.2)	25.2 (3.1)	31.4 (1.2)	26.3 (2.0)
≥$75k	34.2 (0.9)	36.3 (1.0)	29.3 (1.8)	25.4 (1.7)	20.2 (3.4)	21.3 (1.5)	13.9 (2.0)
Health insurance, %	81.1 (0.5)	80.0 (0.5)	80.9 (1.2)	81.3 (1.0)	79.7 (2.2)	95.6 (0.5)	97.0 (0.8)
Employed, %	64.7 (0.5)	69.4 (0.6)	58.0 (1.5)	51.1 (1.4)	43.5 (3.1)	26.1 (1.3)	11.5 (1.7)
**Smoking status, %**							
Never	54.3 (0.5)	54.1 (0.5)	68.4 (1.3)	48.3 (1.2)	61.7 (3.2)	52.7 (1.5)	52.0 (2.5)
Former	23.9 (0.4)	23.0 (0.4)	19.0 (1.2)	26.0 (1.1)	21.4 (2.6)	37.0 (1.4)	40.6 (2.6)
Current	21.9 (0.4)	22.9 (0.5)	12.6 (0.9)	25.7 (1.1)	16.9 (2.8)	10.3 (0.9)	7.4 (1.5)
**Alcohol use, %**							
<1 drink/wk	59.8 (0.7)	58.2 (0.7)	71.9 (1.5)	62.8 (1.6)	71.7 (3.8)	70.2 (1.6)	76.9 (2.6)
1–3 drinks/wk	21.4 (0.4)	22.5 (0.5)	16.4 (1.4)	18.5 (1.2)	12.0 (2.6)	13.1 (1.2)	9.3 (1.9)
≥3 drinks/wk	18.8 (0.5)	19.4 (0.6)	11.7 (1.2)	18.7 (1.3)	16.3 (3.5)	16.7 (1.3)	13.8 (2.2)
BMI, kg/m^2^	28.8 (0.1)	28.6 (0.1)	29.6 (0.2)	30.3 (0.3)	29.9 (0.5)	29.4 (0.2)	30.0 (0.5)
CHD, %	3.2 (0.1)	2.3 (0.1)	2.8 (0.5)	5.1 (0.5)	11.1 (2.8)	13.3 (1.0)	17.6 (2.2)
Angina, %	2.4 (0.1)	1.7 (0.1)	2.2 (0.4)	3.4 (0.4)	7.8 (2.4)	9.1 (0.9)	10.8 (2.0)
MI, %	3.2 (0.1)	2.3 (0.1)	2.7 (0.4)	5.0 (0.4)	8.2 (2.1)	13.1 (0.9)	19.9 (1.6)
Stroke, %	2.5 (0.1)	1.6 (0.1)	2.7 (0.4)	4.5 (0.4)	5.8 (1.5)	10.0 (0.7)	18.3 (1.9)
CHF, %	2.1 (0.1)	1.1 (0.1)	2.4 (0.4)	4.2 (0.4)	6.2 (1.5)	9.8 (0.7)	21.5 (1.9)
COPD, %	6.7 (0.2)	6.0 (0.2)	7.0 (0.7)	10.3 (0.8)	7.8 (1.4)	11.0 (0.9)	15.5 (1.9)
Diabetes, %	12.6 (0.3)	9.4 (0.2)	15.3 (1.0)	29.6 (1.4)	36.2 (3.0)	29.5 (1.3)	46.2 (2.8)
Hypertension, %	36.6 (0.5)	31.7 (0.5)	37.0 (1.5)	56.0 (1.3)	60.2 (3.1)	75.2 (1.2	90.1 (1.2)
Hyperlipidemia, %	67.8 (0.4)	66.5 (0.5)	54.4 (1.5)	76.0 (1.2	66.3 (3.4)	87.3 (0.9)	83.1 (1.8)
Arthritis, %	24.2 (0.4)	21.3 (0.4)	26.5 (1.4)	32.6 (1.2)	34.4 (3.0)	50.4 (1.6)	59.1 (2.6)
Hemoglobin (g/dl)	14.3 (0.0)	14.6 (0.0)	11.3 (0.0)	14.5 (0.0)	11.3 (0.1)	14.1 (0.0)	11.4 (0.0)
Iron (*µ*g/dl)	87.0 (0.3)	89.8 (0.3)	51.7 (1.2)	85.5 (1.0)	53.2 (2.0)	82.0 (0.8)	65.3 (1.6)
Ferritin (ng/ml)	82.7 (1.6)	81.6 (1.6)	45.5 (4.8)	106.2 (7.7)	62.3 (10.4)	136.9 (9.0)	120.9 (14.2)
eGFR (ml/min per 1.73 m^2^)	94.8 (0.3)	97.8 (0.3)	101.5 (0.6)	95.4 (0.5)	95.2 (1.3)	49.9 (0.2)	41.6 (0.6)
ACR	29.1 (1.1)	7.5 (0.1)	9.1 (0.2)	168.5 (8.8)	252.7 (41.7)	110.2 (13.4)	341.3 (40.8)

Anemia status was classified using the World Health Organization criteria (hemoglobin levels <12 g/dl in women and <13 g/dl in men); CKD was classified using the Kidney Disease Outcomes Quality Initiative 2002 criteria (CKD stage 1, albuminuria and eGFR ≥90 ml/min per 1.73 m^2^; CKD stage 2, albuminuria and eGFR 60–89 ml/min per 1.73 m^2^; CKD stage 3, eGFR 30–59 ml/min per 1.73 m^2^; CKD stage 4, eGFR 15–29 ml/min per 1.73 m^2^; CKD stage 5, eGFR <15 ml/min per 1.73 m^2^). GED, general educational development; AA, associate of arts; BMI, body mass index; CHD, coronary heart disease; MI, myocardial infarction; CHF, chronic heart failure; COPD, chronic obstructive pulmonary disease; ACR, albumin-creatinine ratio.

Values in the table are mean (SEM) or percentage (SEM).

Data were available on all sample participants (*N*=33,300), except for education (data available in 33,266), marital status (data available in 33,140 participants), house income (data available in 30,433 participants), health insurance (data available in 33,206 participants), employment (data available in 33,283 participants), smoking status (data available in 33,277 participants), alcohol use (data available in 26,144 participants).

The mean (SEM) overall, lower extremity mobility, general physical activity, ADL, IADLs, and leisure and social activities scores were 4.0 (0.1), 7.8 (0.2), 5.0 (0.1), 1.8 (0.1), 3.0 (0.1), and 2.9 (0.1), respectively (Table 2). Stooping, crouching, or kneeling and standing for long periods had highest scores (9.9 and 9.6, respectively), while using fork, knife, drinking cup, and difficulties on leisure activities at home had the lowest (0.8 and 1.3, respectively). All these scores increased with increasing category of CKD severity and with the presence of anemia. When the responses to physical functioning questions were categorized between no difficulty and some difficulty, much difficulty, or unable to do, the proportion of participants with at least some difficult for any activity and for lower extremity mobility, general physical activity, ADL, IADLs, and leisure and social activities were 27.8 (0.5), 21.9 (0.4), 22.8 (0.5), 10.3 (0.3), 12.9 (0.3), and 10.0% (0.3), respectively (Table 3).

**Table 2.** Mean scores for activities of daily living by CKD category and anemia status, National Health and Nutrition Survey 1999–2016

Range (0–100)	Overall	No CKD		CKD Stages 1–2		CKD Stages 3–5	
		No Anemia	Anemia	No Anemia	Anemia	No Anemia	Anemia
Overall ADL score	4.0 (0.1)	3.1 (0.1)	4.8 (0.3)	6.6 (0.3)	8.9 (0.9)	9.8 (0.4)	16.4 (0.9)
**Lower extremity mobility**							
Overall	7.8 (0.2)	6.0 (0.2)	9.1 (0.6)	13.3 (0.6)	17.6 (1.8)	21.6 (0.8)	33.8 (1.8)
Walking for a quarter mile	4.2 (0.2)	3.2 (0.1)	5.1 (0.5)	7.1 (0.6)	10.1 (2.0)	14.0 (0.8)	24.4 (1.9)
Walking ten steps	2.8 (0.1)	2.1 (0.1)	3.8 (0.4)	5.1 (0.4)	7.6 (1.5)	8.8 (0.6)	16.1 (2.0)
Stooping, crouching, or kneeling	9.9 (0.2)	8.0 (0.2)	10.8 (0.7)	16.1 (0.7)	19.9 (1.9)	26.4 (0.9)	35.4 (2.1)
**General physical activity**							
Overall	5.0 (0.1)	4.0 (0.1)	6.0 (0.4)	8.3 (0.3)	10.6 (1.1)	12.3 (0.5)	20.3 (0.9)
Lifting or carrying	5.2 (0.2)	4.0 (0.1)	6.7 (0.6)	9.5 (0.6)	13.2 (1.8)	13.5 (0.7)	23.9 (1.6)
Standing up from armless chair	3.8 (0.1)	2.9 (0.1)	4.6 (0.4)	6.2 (0.3)	9.5 (1.1)	10.7 (0.6)	18.8 (1.5)
Standing for long periods	9.6 (0.2)	7.5 (0.2)	11.0 (0.7)	16.3 (0.7)	19.8 (2.2)	26.9 (1.0)	44.7 (1.8)
Sitting for long periods	5.0 (0.2)	4.5 (0.2)	6.0 (0.5)	7.3 (0.4)	6.5 (0.9)	8.1 (0.5)	11.5 (1.2)
Reaching up over head	3.5 (0.1)	2.9 (0.1)	4.1 (0.4)	5.8 (0.4)	8.3 (1.4)	7.8 (0.5)	12.7 (1.4)
Grasp/holding small objects	2.8 (0.1)	2.2 (0.1)	3.1 (0.3)	4.5 (0.3)	6.0 (1.3)	6.8 (0.4)	10.5 (1.0)
**ADL**							
Overall	1.8 (0.1)	1.4 (0.1)	2.6 (0.2)	3.1 (0.2)	4.5 (0.5)	4.0 (0.2)	7.7 (0.7)
Walking between rooms same floor	1.4 (0.1)	1.0 (0.0)	2.0 (0.2)	2.6 (0.2)	4.0 (0.7)	4.4 (0.4)	9.1 (1.2)
Getting in and out of bed	2.9 (0.1)	2.5 (0.1)	3.9 (0.4)	4.8 (0.3)	7.0 (0.9)	5.5 (0.4)	8.9 (1.0)
Using fork, knife, drinking cup	0.8 (0.0)	0.7 (0.0)	1.3 (0.2)	1.6 (0.2)	1.9 (0.6)	1.7 (0.2)	3.6 (0.6)
Dressing yourself	2.1 (0.1)	1.7 (0.1)	3.2 (0.3)	3.5 (0.3)	5.0 (0.8)	4.3 (0.3)	9.2 (1.0)
**IADLs**							
Overall	3.0 (0.1)	2.4 (0.1)	3.6 (0.3)	4.8 (0.3)	7.0 (0.8)	7.3 (0.4)	12.3 (1.0)
Managing money	2.4 (0.1)	2.1 (0.1)	2.5 (0.3)	3.6 (0.3)	4.3 (0.8)	4.3 (0.4)	6.2 (1.0)
House chore	4.5 (0.1)	3.6 (0.1)	5.5 (0.4)	7.7 (0.4)	10.9 (1.3)	12.0 (0.6)	19.7 (1.3)
Preparing meals	1.7 (0.1)	1.3 (0.1)	2.2 (0.3)	2.7 (0.3)	5.0 (0.9)	4.9 (0.4)	9.3 (1.1)
**Leisure and social activities**							
Overall	2.9 (0.1)	2.3 (0.1)	3.4 (0.3)	4.9 (0.3)	6.8 (1.1)	6.4 (0.4)	12.2 (1.1)
Going out to movies, events	3.9 (0.1)	3.1 (0.1)	4.9 (0.5)	6.8 (0.4)	8.2 (1.2)	9.4 (0.5)	17.1 (1.5)
Attending social event	3.1 (0.1)	2.5 (0.1)	3.5 (0.4)	5.3 (0.4)	9.1 (2.0)	6.7 (0.4)	14.6 (1.4)
Leisure activity at home difficulty	1.3 (0.1)	1.1 (0.1)	1.4 (0.2)	2.2 (0.2)	2.6 (0.7)	2.6 (0.3)	4.1 (0.7)

Anemia status was classified using the World Health Organization criteria (hemoglobin levels <12 g/dl in women and <13 g/dl in men); CKD was classified using the Kidney Disease Outcomes Quality Initiative 2002 criteria (CKD stage 1, albuminuria and eGFR ≥90 ml/min per 1.73 m^2^; CKD stage 2, albuminuria and eGFR 60–89 ml/min per 1.73 m^2^; CKD stage 3, eGFR 30–59 ml/min per 1.73 m^2^; CKD stage 4, eGFR 15–29 ml/min per 1.73 m^2^; CKD stage 5, eGFR <15 ml/min per 1.73 m^2^). ADL, activities of daily living; IADL, instrumental ADL.

Scores for overall and individual activities of daily livings ranged from 0 to 100. Higher scores reflect worse function.

Values in the table are mean (SEM).

**Table 3.** Percentage of participants with difficulties in activities of daily living by CKD category and anemia status, National Health and Nutrition Survey 1999–2016

Variable	Overall	No CKD		CKD Stages 1–2		CKD Stages 3–5	
		No Anemia	Anemia	No Anemia	Anemia	No Anemia	Anemia
Overall ADL score	27.8 (0.5)	23.6 (0.5)	29.9 (1.4)	40.9 (1.3)	47.6 (3.3)	64.2 (1.4)	81.2 (1.9)
**Lower extremity mobility**							
Overall	21.9 (0.4)	18.0 (0.4)	23.8 (1.3)	33.7 (1.3)	41.5 (3.3)	55.7 (1.4)	70.0 (2.8)
*Walking for a quarter mile*	8.8 (0.3)	7.0 (0.3)	10.1 (0.9)	14.6 (1.1)	18.1 (2.7)	26.0 (1.3)	43.9 (3.1)
*Walking ten steps*	6.4 (0.2)	4.9 (0.2)	8.8 (0.9)	11.8 (1.0)	15.2 (2.5)	19.0 (1.2)	31.4 (3.2)
*Stooping, crouching, or kneeling*	19.8 (0.4)	16.3 (0.4)	21.2 (1.3)	30.3 (1.3)	37.9 (3.2)	50.1 (1.3)	61.4 (3.0)
**General physical activity**							
Overall	22.8 (0.5)	19.0 (0.5)	24.9 (1.5)	34.2 (1.2)	41.5 (3.2)	54.9 (1.5)	75.2 (1.8)
*Lifting or carrying*	9.8 (0.3)	7.8 (0.3)	12.5 (1.0)	16.7 (0.9)	22.1 (2.7)	24.2 (1.1)	40.2 (2.4)
*Standing up from armless chair*	9.0 (0.2)	7.1 (0.2)	10.2 (0.9)	14.5 (0.7)	22.0 (2.4)	24.3 (1.3)	40.1 (2.7)
*Standing for long periods*	17.1 (0.4)	13.8 (0.4)	19.4 (1.2)	26.9 (1.1)	31.8 (3.2)	44.4 (1.4)	63.4 (2.0)
*Sitting for long periods*	10.7 (0.3)	9.5 (0.3)	12.7 (1.0)	15.6 (0.7)	15.5 (2.2)	18.3 (1.0)	24.8 (2.1)
*Reaching up over head*	7.7 (0.2)	6.4 (0.2)	8.8 (0.7)	12.9 (0.8)	16.5 (2.4)	16.4 (0.9)	25.1 (2.1)
*Grasp/holding small objects*	6.9 (0.2)	5.6 (0.2)	7.5 (0.8)	11.1 (0.8)	13.8 (2.6)	16.4 (1.0)	24.7 (2.2)
**ADL**							
Overall	10.3 (0.3)	8.4 (0.3)	13.0 (1.0)	16.6 (0.8)	24.2 (2.7)	22.2 (1.1)	38.4 (2.7)
*Walking between rooms (same floor)*	3.5 (0.1)	2.6 (0.1)	4.9 (0.6)	6.3 (0.5)	8.7 (1.5)	10.0 (0.8)	19.2 (2.1)
*Getting in and out of bed*	7.4 (0.2)	6.3 (0.2)	9.4 (0.9)	12.0 (0.8)	17.0 (2.3)	13.8 (0.9)	22.1 (2.2)
*Using fork, knife, drinking from cup*	2.2 (0.1)	1.7 (0.1)	3.3 (0.5)	4.0 (0.5)	5.2 (1.8)	4.1 (0.5)	9.4 (1.4)
*Dressing yourself*	5.3 (0.2)	4.3 (0.2)	7.7 (0.7)	8.8 (0.6)	11.3 (1.7)	11.1 (0.9)	21.5 (2.0)
**IADLs**							
Overall	12.9 (0.3)	10.8 (0.3)	15.1 (0.9)	20.7 (1.0)	28.7 (3.0)	27.2 (1.2)	39.6 (2.5)
*Managing money*	5.1 (0.2)	4.5 (0.2)	5.4 (0.6)	8.0 (0.7)	9.7 (2.0)	8.4 (0.7)	10.9 (1.5)
*House chore*	9.9 (0.3)	8.0 (0.3)	12.0 (0.8)	16.7 (0.8)	23.7 (2.8)	23.9 (1.1)	37.8 (2.5)
*Preparing meals*	3.8 (0.1)	3.0 (0.1)	4.6 (0.6)	5.6 (0.6)	10.3 (1.8)	10.2 (0.7)	17.7 (2.0)
**Leisure and social activities**							
Overall	10.0 (0.3)	8.3 (0.3)	11.4 (0.8)	17.1 (0.9)	20.8 (2.8)	21.2 (1.0)	34.3 (2.4)
*Going out to movies, events*	8.4 (0.2)	6.8 (0.2)	9.8 (0.8)	15.0 (0.8)	15.6 (2.4)	18.9 (0.9)	31.6 (2.6)
*Attending social event*	6.7 (0.2)	5.5 (0.2)	7.8 (0.8)	11.5 (0.7)	17.1 (2.8)	13.3 (0.8)	26.3 (2.3)
*Leisure activity at home difficulty*	3.3 (0.1)	2.8 (0.1)	3.6 (0.5)	5.5 (0.5)	6.8 (2.0)	6.3 (0.7)	9.4 (1.4)

Anemia status was classified using the World Health Organization criteria (hemoglobin levels <12 g/dl in women and <13 g/dl in men); CKD was classified using Kidney Disease Outcomes Quality Initiative 2002 criteria (CKD stage 1, albuminuria and eGFR ≥90 ml/min per 1.73 m^2^; CKD stage 2, albuminuria and eGFR 60–89 ml/min per 1.73 m^2^; CKD stage 3, eGFR 30–59 ml/min per 1.73 m^2^; CKD stage 4, eGFR 15–29 ml/min per 1.73 m^2^; CKD stage 5, eGFR <15 ml/min per 1.73 m^2^). ADL, activities of daily living; IADL, instrumental ADL.

Participants with difficulties in activities of daily living were defined as those with some difficulty, much difficulty, or unable to do the activity (scores 1–3).

Values in the table are percentage (SEM).

**Figure 2.**
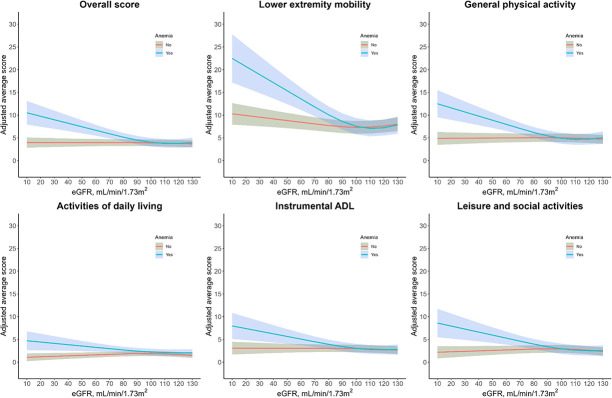
**Adjusted mean differences (95% CI) for functional and ADL scores by eGFR, National Health and Nutrition Survey 1999–2016.** Adjusted for age, sex, race/ethnicity, survey cycle, education, marital status, household income, health insurance, maintained employment, smoking status, alcohol use, comorbidities (congestive heart failure, CHD, angina pectoris, heart attack, stroke, arthritis, COPD), serum iron, urine ACR, BMI, hypertension, diabetes, and hyperlipidemia. ACR, albumin-creatinine ratio; ADL, activities of daily living; BMI, body mass index; CHD, coronary heart disease; CI, confidence interval; COPD, chronic obstructive pulmonary disease.

In multivariable adjusted analyses, participants with anemia had significantly higher (worse) overall physical functioning scores among all participants with CKD (Figure 2 and Table 4). The adjusted mean differences (95% confidence interval [CI]) in overall score comparing participants with anemia with those without anemia among participants with no CKD, CKD stages 1–2, and stages 3–5 were 0.6 (−0.1 to 1.0), 1.5 (0.2 to 2.8), and 3.7 (2.1 to 5.3). By domain, participants with anemia had significantly higher (worse) physical functioning scores for ADLs among participants with no CKD and CKD stages 1–2 and for all domains among participants with CKD stages 3–5. Anemia and CKD showed a supra-additive interaction for all physical functioning outcomes among participants in CKD stages 3–5 (Table 4). The conclusions were consistent when we used the prevalence of participants with at least some difficulty in physical functioning activities as the outcome variable (Supplemental Table 1).

**Table 4.** Adjusted mean differences (95% confidence interval) in activities of daily living scores comparing participants with and without anemia by CKD category, National Health and Nutrition Survey 1999–2016

Variable	No CKD		CKD Stages 1–2		CKD Stages 3–5	
	No Anemia	Anemia	No Anemia	Anemia	No Anemia	Anemia
**Overall ADL score**						
Model 1	Ref	1.0 (0.4 to 1.7)^a^	Ref	1.8 (0.1 to 3.5)^a^	Ref	6.5 (4.7 to 8.3)^b^
Model 2	Ref	0.5 (−0.0 to 1.1)	Ref	1.5 (0.2 to 2.7)^a^	Ref	3.9 (2.2 to 5.6)^b^
Model 3	Ref	0.5 (−0.1 to 1.0)	Ref	1.5 (0.2 to 2.8)^a^	Ref	3.6 (2.0 to 5.2)^b^
**Lower extremity mobility**						
Model 1	Ref	1.9 (0.7 to 3.0)^a^	Ref	3.3 (−0.2 to 6.8)	Ref	11.8 (7.6 to 16.0)^b^
Model 2	Ref	1.0 (−0.0 to 2.0)	Ref	2.8 (−0.1 to 5.7)	Ref	7.0 (3.0 to 11.0)^b^
Model 3	Ref	0.8 (−0.2 to 1.8)	Ref	2.9 (0.1 to 5.7)^a^	Ref	6.4 (2.7 to 10.2)^b^
**General physical activity**						
Model 1	Ref	1.0 (0.2 to 1.8)^a^	Ref	1.6 (−0.4 to 3.6)	Ref	7.8 (5.8 to 9.7)^b^
Model 2	Ref	0.5 (−0.3 to 1.2)	Ref	1.2 (−0.3 to 2.8)	Ref	4.6 (2.8 to 6.5)^b^
Model 3	Ref	0.4 (−0.3 to 1.1)	Ref	1.2 (−0.3 to 2.8)	Ref	4.3 (2.5 to 6.1)^b^
**ADL**						
Model 1	Ref	0.9 (0.4 to 1.4)^a^	Ref	1.1 (0.0 to 2.1)	Ref	3.6 (2.2 to 5.1)^b^
Model 2	Ref	0.6 (0.2 to 1.1)^a^	Ref	0.9 (−0.0 to 1.9)	Ref	2.2 (0.9 to 3.6)^b^
Model 3	Ref	0.6 (0.1 to 1.0)^a^	Ref	0.9 (−0.0 to 1.8)^a^	Ref	2.0 (0.7 to 3.3)^b^
**IADL**						
Model 1	Ref	0.6 (0.1 to 1.2)^a^	Ref	1.8 (0.2 to 3.4)^a^	Ref	4.9 (2.8 to 7.0)^b^
Model 2	Ref	0.3 (−0.3 to 0.8)	Ref	1.5 (−0.0 to 2.9)	Ref	2.7 (0.6 to 4.8)^b^
Model 3	Ref	0.2 (−0.4 to 0.7)	Ref	1.5 (−0.0 to 2.9)	Ref	2.6 (0.5 to 4.7)^b^
**Leisure and social activities**						
Model 1	Ref	0.8 (0.1 to 1.4)^a^	Ref	1.6 (−0.7 to 4.0)	Ref	5.8 (3.7 to 8.0)^b^
Model 2	Ref	0.4 (−0.2 to 1.0)	Ref	1.3 (−0.8 to 3.4)	Ref	3.7 (1.6 to 5.8)^b^
Model 3	Ref	0.3 (−0.3 to 1.0)	Ref	1.3 (−0.8 to 3.4)	Ref	3.5 (1.4 to 5.6)^b^

Anemia status was classified using the World Health Organization criteria (hemoglobin levels <12 g/dl in women and <13 g/dl in men); CKD was classified using the Kidney Disease Outcomes Quality Initiative 2002 criteria (CKD stage 1, albuminuria and eGFR ≥90 ml/min per 1.73 m^2^; CKD stage 2, albuminuria and eGFR 60–89 ml/min per 1.73 m^2^; CKD stage 3, eGFR 30–59 ml/min per 1.73 m^2^; CKD stage 4, eGFR 15–29 ml/min per 1.73 m^2^; CKD stage 5, eGFR <15 ml/min per 1.73 m^2^). ADL, activities of daily living; IADL, instrumental ADL.

Model 1 adjusted for age, sex, race/ethnicity, and survey cycle.

Model 2 further adjusted for education, marital status, household income, health insurance, maintained employment, smoking status, alcohol use, and comorbidities (congestive heart failure, coronary heart disease, angina pectoris, heart attack, stroke, arthritis, chronic obstructive pulmonary disease).

Model 3 further adjusted for serum iron, urine albumin-creatinine ratio, body mass index, hypertension, diabetes, and hyperlipidemia.

a Youssef MK Farag (Boston, Massachusetts), Myrlene Sanon (Princeton, New Jersey)

b *P* value for interaction of CKD category by anemia group <0.05.

### Physical Activity

For the physical activity analysis (NHANES 2007–2016), 22,933 participants were eligible and had complete data (Supplemental Figure 1). The characteristics of these participants are shown in (Supplemental Table 2). The mean weekly MET-minute score decreased with declining renal function and with the presence of anemia (Supplemental Table 3). Similarly, the proportion of participants with high physical activity decreased with declining renal function and with the presence of anemia (Supplemental Table 4).

In multivariable adjusted analyses, the adjusted mean difference in weekly MET-minute score was significantly lower in participants with anemia compared with those without anemia among participants in CKD stages 3–5 (mean difference, −501.3 MET-minutes; 95% CI, −803.9 to −198.7; Supplemental Table 5). The adjusted prevalence of high physical activity was also significantly lower in participants with anemia compared with those without anemia among participants in CKD stages 3–5 (adjusted prevalence ratio, 0.70; 95% CI, 0.51 to 0.96; Supplemental Table 6).

## Discussion

In a large sample representative of the US adult, noninstitutionalized population, physical functioning was worse in patients with anemia compared with those without anemia among participants with CKD. The physical function associated with anemia was worst in participants with CKD stages 3–5, for whom all domain scores were significantly worse in participants with compared with those without anemia. Among participants with CKD stages 3–5, anemia was also associated with a significantly lower level of physical activity. Overall, our data support the hypothesis that the combination of CKD and anemia has a significant effect on physical functioning above and beyond the independent effect of each condition. Importantly, we defined anemia as hemoglobin <12 g/dl in women and <13 g/dl in men using the World Health Organization criteria.^19^ We did not have enough cases of severe anemia to allow for more detailed subgroup analyses, but our findings indicate that even moderate levels of anemia is associated with suboptimal physical functioning in participants with CKD.

Previous studies have also identified an association between CKD and self-reported physical functioning and physical activity.^26–28^ Using data from NHANES 1999–2006 (*N*=16,011), the Centers for Disease Control CKD Surveillance Team^27^ found that CKD was associated with a higher prevalence of reported limitations in working, walking, and cognition, with increased difficulties in total ADLs, IADLs, and leisure and social activities. The Korean Longitudinal Study on Health and Aging,^28^ conducted in a randomly selected community-based older population (*N*=984), found that decreasing renal function was significantly associated with increased (worse) ADL/IADL scores and that increased ADL/IADL scores were associated with increased all-cause mortality among participants with CKD. With respect to physical activity, a study using data from NHANES III (1988–1994; *N*=15,368 participants) found that CKD was associated with an increased prevalence of inactive participants (29.0% versus 14.0%).^29^ A study in Taiwan, however, found no association between CKD and the prevalence of inactive participants.^30^ These studies, however, did not examine the joint association of CKD and anemia with the prevalence of limitations in physical functioning or physical activity.

The few studies that have assessed the association between anemia and functional outcomes have been restricted to nondialysis patients with CKD stages 3 or greater^8,31–33^ or restricted to hemodialysis patients.^34^ As expected from studies in CKD populations, in all these studies, the mean hemoglobin levels were lower, and the proportion of participants with moderate-to-severe anemia (hemoglobin <10 g/dl) was higher than in our sample. The CKD Outcomes and Practice Patterns Study,^8^ a prospective cohort of 2465 patients with stage 3–5 CKD, found that anemia was associated with lower (worse) overall health-related quality of life and with the emotional and physical domains of health-related quality of life, as well as with physical inactivity. Similar findings were obtained in the CKD RenalSoft Informatics Observational Study,^31^ a prospective cohort of 1186 patients with stage 3–5 CKD, that assessed physical functioning using the Kidney Disease Quality of Life Short Form Questionnaire and in the Chinese Cohort Study of CKD,^32^ a multicenter prospective cohort of 2921 patients with CKD. Furthermore, a large real-world cross-sectional multicounty survey of 5276 patients with CKD^33^ found that lower hemoglobin levels worsened the effect of CKD on health-related quality of life. Finally, the Dialysis Outcomes and Practice Patterns Study,^34^ a prospective cohort of 5763 hemodialysis patients, did not find a consistent association between hemoglobin levels and physical activity.

CKD is associated with increased risk of cardiovascular disease^35^ and with multiple metabolic abnormalities, including anemia,^36,37^ inflammation,^37,38^ oxidative stress,^39^ volume overload and electrolyte abnormalities,^40^ disordered mineral metabolism,^41^ and uremic toxin accumulation,^42^ that further contribute to frailty, decreased physical function, and decreased quality of life.^26^ In our analysis, even in the absence of anemia, the percentage of participants with difficulties in ADL was much higher in patients with CKD stages 1–2 versus no CKD (40.9% versus 23.6%), indicating that the decline of physical function may not only occur in advanced CKD but could start at early CKD stages. ADL assessment among patients with moderate decrease of eGFR could potentially identify patients with physical dysfunction that may be still ameliorable by early interventions.

Anemia is associated with disability, immobility, decreased physical performance, and reduced muscle strength,^43^ as well as with an increased risk of falls and fractures,^44^ but the interactive effect of anemia and CKD on physical function, mental health, and health status in the general population has not been evaluated. Our analysis extends the findings of previous studies and shows that CKD and anemia are associated with a higher compromise of physical functioning than would be expected from the independent association with each condition. Furthermore, this interaction happens with relatively mild levels of anemia and moderate eGFR as we observed in our general population sample. Indeed, after adjustment for demographic risk factors and comorbidities, the ADL scores differed by anemia status at eGFR of as high as 90 ml/min per 1.73 m^2^, which is within the normal range.

Although worse physical function was observed among patients with both anemia and kidney disease, anemia treatment did not result in improved physical function in several clinical trials. In the Correction of Hemogloblin and Outcomes in Renal Insufficiency trial of 1432 patients with CKD, epoetin alfa that targeted hemoglobin level of 13.5 versus 11.3 g/dl in control group did not improve physical function.^18^ In the Trial to Reduce Cardiovascular Events with Aranesp Therapy involving 4038 patients with diabetes, CKD, and anemia, change in physical function did not differ significantly between patients in darbepoetin alfa (mean change 1.3±9.2) versus control group (mean change 1.1±8.8).^17^ Similarly, the Normal Hematocrit Trial failed to show improved physical function by targeting higher hematocrit levels among 1265 hemodialysis patients.^16^ It is possible that there is a threshold effect of increasing hemoglobin on physical function improvement. It is also possible that anemia is a risk mediator for other comorbidities that cause poorer physical function together with CKD, and treating anemia alone is insufficient in improving physical function in patients with CKD.

The strengths of our analysis include the use of a nationally representative sample, the large sample size, and the high quality of the field procedures and laboratory methods of NHANES. There are also several limitations to this study. First, the cross-sectional design does not allow to establish the temporal relationship between CKD and the deterioration of physical functioning and reduction in physical activity. In addition, the associations do not establish a causal effect of CKD or anemia on physical function and physical activity. Second, the results may be due to unmeasured confounders, and the supra-additive associations could be the results of other factors such as diseases, certain medications, or misclassification of CKD severity. For example, we were unable to account for iron deficiency as a confounder because serum ferritin and transferrin saturation were only measured in a small subsample of study participants. Third, the use of single exposure and outcome assessments and the use of self-report to assess physical functioning and physical activity may lead to misclassification and to reduced measures of association. Fourth, ADL in this study was defined differently from other studies that used the Katz-ADL index score^45^ or the Lawton IADL scale.^46^ As a result, the adjusted mean differences in ADL scores in this study may not be directly comparable with other studies, and the clinical interpretation of the differences in ADL scores needs to take into consideration the variation in definitions. Finally, the number of participants with very advanced CKD (stage 4 or 5) was relatively small, and while our findings are representative of the general population, they may not extrapolate to kidney disease clinic or to hemodialysis center patient populations.

In summary, we found that CKD and anemia were associated with impairments in physical functioning and with reduced physical activity. For physical functioning outcomes, the presence of CKD and anemia showed a positive interaction, and their combined effect was higher than that expected from their independent effects. These findings highlight the importance of anemia as a determinant of physical functioning and quality of life in patients with CKD. Clinical management of anemia may be needed to improve physical functioning and physical activity in patients with CKD.

## Supplementary Material

## Supplemental Material

Supplemental Table 1. Adjusted prevalence ratios (95% CI) for difficulties in activities of daily living comparing participants with and without anemia by CKD categories, National Health and Nutrition Survey 1999–2016.

Supplemental Table 2. Characteristics of study participants with physical activity information, National Health and Nutrition Survey 2007–2016.

Supplemental Table 3. Weekly MET-minute score by CKD and anemia status, National Health and Nutrition Survey 2007–2016.

Supplemental Table 4. Percentage of participants with low (<500 MET-minute per week), moderate (500–1000 MET-minute per week), and high (>1000 MET-minute per week) physical activity by CKD and anemia status, National Health and Nutrition Survey 2007–2016.

Supplemental Table 5. Adjusted mean differences (95% CI) in weekly MET-minute score comparing participants with and without anemia by CKD categories, National Health and Nutrition Survey 2007–2016.

Supplemental Table 6. Adjusted prevalence rate ratios (95% CI) by weekly MET-minute score category comparing participants with and without anemia by CKD categories, National Health and Nutrition Survey 2007–2016.

Supplemental Figure 1. Flowchart of study participants in the physical activity component, National Health and Nutrition Survey 2007–2016.
