# Supplementary material for: Effect of Anemia on Physical Function and Physical Activity in CKD: The National Health and Nutrition Examination Survey, 1999–2016
Source: Kidney360. 2023 Sep 28;4(9):e1212–22. doi: 10.34067/KID.0000000000000218 (PMC10550006; doi:10.34067/KID.0000000000000218)

SUPPLEMENTAL MATERIAL

Impact of anemia on physical function and physical activity in chronic kidney disease: The National Health and Nutrition Examination Survey, 1999–2016

Youssef M. Farag, MD, PhD, Elena Blasco-Colmenares, MD, PhD, Di Zhao, PhD,
Efstathios N. Michalopoulos, Myrlene Sanon, MPH, Eliseo Guallar MD, DrPH
Fredric O. Finkelstein, MD

Supplemental Table 1. Adjusted prevalence ratios (95% CI) for difficulties in activities of daily living comparing participants with and without anemia by chronic kidney disease categories, National Health and Nutrition Survey 1999–2016.

Supplemental Table 2. Characteristics of study participants with physical activity information, National Health and Nutrition Survey 2007–2016.

Supplemental Table 3. Weekly MET-minute score by chronic kidney disease and anemia status, National Health and Nutrition Survey 2007–2016.

Supplemental Table 4. Percentage of participants with low (< 500 MET-minute per week), moderate (500 to 1000 MET-minute per week) and high (> 1000 MET-minute per week) physical activity by chronic kidney disease and anemia status, National Health and Nutrition Survey 2007–2016.

Supplemental Table 5. Adjusted mean differences (95% CI) in Weekly MET-minute score comparing participants with and without anemia by chronic kidney disease categories, National Health and Nutrition Survey 2007–2016.

Supplemental Table 6. Adjusted prevalence rate ratios (95% CI) by Weekly MET-minute score category comparing participants with and without anemia by chronic kidney disease categories, National Health and Nutrition Survey 2007 –2016.

Supplemental Figure 1. Flowchart of study participants in the physical activity component, National Health and Nutrition Survey 2007–2016.

Supplemental Table 1. Adjusted prevalence ratios (95% CI) for difficulties in activities of daily living comparing participants with and without anemia by chronic kidney disease categories, National Health and Nutrition Survey 1999–2016.

	**No CKD**		**CKD stages 1–2**		**CKD stages 3–5**	
	**No anemia**	**Anemia**	**No anemia**	**Anemia**	**No anemia**	**Anemia**
**Overall ADL score**						
Model 1	Ref	**1.2 (1.1, 1.3)**	Ref	1.1 (1.0, 1.3)	Ref	**1.5 (1.3, 1.7)***
Model 2	Ref	**1.1 (1.0, 1.2)**	Ref	1.1 (1.0, 1.3)	Ref	**1.2 (1.1, 1.4)***
Model 3	Ref	**1.1 (1.0, 1.2)**	Ref	**1.1 (1.0, 1.3)**	Ref	**1.2 (1.1, 1.4)**
**Lower extremity mobility**						
Model 1	Ref	**1.2 (1.1, 1.3)**	Ref	1.2 (1.0, 1.4)	Ref	**1.4 (1.2, 1.7)**
Model 2	Ref	1.1 (1.0, 1.2)	Ref	**1.2 (1.0, 1.4)**	Ref	1.1 (1.0, 1.4)
Model 3	Ref	1.1 (1.0, 1.2)	Ref	**1.2 (1.1, 1.4)**	Ref	1.1 (0.9, 1.3)
**General physical activity**						
Model 1	Ref	**1.2 (1.1, 1.3)**	Ref	1.2 (1.0, 1.4)	Ref	**1.6 (1.4, 1.8)***
Model 2	Ref	1.1 (1.0, 1.2)	Ref	**1.2 (1.0, 1.4)**	Ref	**1.3 (1.2, 1.5)***
Model 3	Ref	1.1 (1.0, 1.2)	Ref	**1.2 (1.0, 1.4)**	Ref	**1.3 (1.2, 1.5)***
**Activities of daily living**						
Model 1	Ref	**1.3 (1.2, 1.6)**	Ref	**1.3 (1.0, 1.8)**	Ref	**1.8 (1.5, 2.2)***
Model 2	Ref	**1.2 (1.1, 1.4)**	Ref	**1.4 (1.1, 1.7)**	Ref	**1.4 (1.2, 1.7)**
Model 3	Ref	**1.1 (1.0, 1.3)**	Ref	**1.4 (1.1, 1.7)**	Ref	**1.3 (1.1, 1.6)**
**Instrumental activities of daily living**						
Model 1	Ref	**1.2 (1.1, 1.4)**	Ref	**1.3 (1.0, 1.6)**	Ref	**1.5 (1.3, 1.9)***
Model 2	Ref	1.1 (1.0, 1.2)	Ref	**1.3 (1.1, 1.6)**	Ref	1.2 (1.0, 1.5)
Model 3	Ref	1.1 (1.0, 1.2)	Ref	**1.3 (1.1, 1.7)**	Ref	1.2 (1.0, 1.4)

Leisure and social activities						
Model 1	Ref	**1.2 (1.0, 1.4)**	Ref	1.1 (0.9, 1.5)	Ref	**1.7 (1.4, 2.0)***
Model 2	Ref	1.1 (0.9, 1.2)	Ref	1.1 (0.9, 1.4)	Ref	**1.3 (1.1, 1.6)**
Model 3	Ref	1.1 (0.9, 1.2)	Ref	1.2 (0.9, 1.5)	Ref	**1.3 (1.1, 1.6)**

Anemia status was classified using World Health Organization criteria (hemoglobin levels <12 g/dL in women and <13 g/dL in men); CKD was classified using KDOQI 2002 criteria (CKD Stage 1, albuminuria and eGFR ≥ 90 ml/min/1.73 m^2; CKD Stage 2, albuminuria and eGFR 60-89 ml/min/1.73 m^2; CKD Stage 3, eGFR 30-59 ml/min/1.73 m^2; CKD Stage 4, eGFR 15-29 ml/min/1.73 m^2; CKD Stage 5, eGFR < 15 ml/min/1.73 m^2.

Model 1 adjusted for age, sex, race/ethnicity, and survey cycle.

Model 2 further adjusted for education, marital status, household income, health insurance, maintained employment, smoking status, alcohol use and comorbidities (congestive heart failure, coronary heart disease, angina pectoris, heart attack, stroke, arthritis, chronic obstructive pulmonary disease).

Model 3 further adjusted for serum iron, urine albumin to creatinine ratio, body mass index, hypertension, diabetes, and hyperlipidemia.

[*] p-value for interaction of CKD category by anemia group <0.05.

Supplemental Table 2. Characteristics of study participants with physical activity information, National Health and Nutrition Survey 2007–2016.

	Overall	No CKD		CKD stages 1–2		CKD stages 3–5	
		No anemia	Anemia	No Anemia	Anemia	No anemia	Anemia
**N sample**	22,933	17,801	1,461	1,790	279	1,173	429
**N population, *10^6**	184.4	152.2	8.8	12.2	1.3	7.9	2.0
**Age, years**	45.8 (0.3)	43.8 (0.2)	45.7 (0.6)	49.9 (0.5)	52.6 (1.1)	69.0 (0.5)	71.8 (0.6)
**Gender, % male**	49.2 (0.3)	52.0 (0.4)	22.2 (1.6)	44.5 (1.5)	32.1 (3.8)	38.9 (1.9)	41.9 (2.7)
**Race, %**							
Non-Hispanic White	65.5 (1.7)	67.0 (1.6)	38.8 (2.8)	61.0 (2.5)	33.3 (4.3)	80.1 (1.7)	60.4 (3.0)
Non-Hispanic Black	11.1 (0.8)	9.6 (0.7)	32.4 (2.4)	12.4 (1.2)	37.6 (3.6)	7.9 (0.9)	22.2 (2.2)
Mexican American	9.2 (0.9)	9.3 (0.9)	11.3 (1.5)	11.7 (1.3)	12.3 (2.8)	3.5 (0.6)	5.9 (1.3)
Other Hispanic	6.1 (0.6)	6.1 (0.6)	7.9 (0.9)	6.3 (0.9)	9.5 (1.8)	3.8 (0.6)	4.2 (0.8)
Other	8.0 (0.5)	8.1 (0.5)	9.7 (1.2)	8.6 (0.9)	7.4 (1.4)	4.7 (0.8)	7.3 (1.5)
**Married, %**	55.2 (0.7)	55.8 (0.8)	52.1 (1.9)	50.9 (1.7)	50.3 (3.4)	55.6 (1.9)	47.5 (3.3)
**Education, %**							
<9th grade	6.0 (0.3)	5.3 (0.3)	6.6 (0.6)	9.7 (0.8)	17.0 (2.9)	9.1 (0.9)	14.4 (1.8)
9–11th grade	11.2 (0.5)	10.6 (0.5)	12.9 (1.2)	14.2 (0.9	17.2 (3.0)	13.2 (1.2)	17.2 (2.4)
High school or GED	22.2 (0.6)	21.7 (0.6)	21.4 (1.4)	24.8 (1.7)	18.5 (3.1)	25.7 (1.6)	31.8 (2.4)
Some college or AA	31.4 (0.6)	31.8 (0.6)	32.8 (1.6)	29.3 (1.6)	27.5 (3.3)	27.8 (1.7)	27.5 (2.9)
≥College	29.2 (1.1)	30.6 (1.1)	26.3 (2.0)	22.0 (1.9)	19.9 (3.7)	24.2 (2.0)	9.1 (1.5)
**Household income, %**							
<$20k	14.9 (0.6)	13.4 (0.6)	19.7 (1.6)	22.2 (1.4)	28.1 (3.3)	22.8 (1.6)	31.7 (2.8)
$20k–<$35k	16.3 (0.5)	15.4 (0.5)	19.6 (1.5)	19.5 (1.3)	24.8 (3.8)	20.3 (1.3)	25.7 (2.9)
$35k–<$75k	31.4 (0.7)	31.8 (0.8)	29.9 (1.6)	29.6 (1.4)	24.8 (3.6)	30.8 (1.5)	27.0 (2.5)
≥$75k	37.4 (1.2)	39.5 (1.2)	30.8 (2.0)	28.7 (2.2);	22.3 (4.1)	26.1 (2.2)	15.6 (2.7)
**Health insurance, %**	80.4 (0.6)	79.7 (0.7)	79.0 (1.4)	79.0 (1.2)	76.9 (2.7)	94.9 (0.7)	96.1 (1.2)
**Employed, %**	66.4 (0.7)	70.7 (0.7)	58.7 (1.7)	53.4 (1.8)	43.1 (3.5)	29.0 (1.9)	13.1 (2.1)
**Smoking status, %**							

Never	56.4 (0.7)	56.4 (0.7)	68.6 (1.5)	49.9 (1.7)	63.2 (3.8)	52.3 (2.0)	52.3 (3.0)
Former	23.1 (0.5)	22.3 (0.5)	18.7 (1.4)	24.9 (1.4)	20.5 (3.1)	36.8 (1.8)	39.9 (3.4)
Current	20.5 (0.5)	21.3 (0.6)	12.7 (1.0)	25.3 (1.4)	16.3 (3.3)	10.9 (1.2)	7.8 (2.1)
**Alcohol use, %**							
<1 drink/week	58.6 (0.8)	56.8 (0.9)	72.0 (1.7)	62.1 (2.0)	71.0 (4.6)	68.0 (2.1)	79.2 (2.9)
1–3 drinks/week	22.2 (0.5)	23.4 (0.6)	16.2 (1.5)	18.8 (1.6)	11.7 (2.6)	13.9 (1.5)	8.7 (2.5)
≥3 drinks/week	19.3 (0.6)	19.8 (0.7)	11.8 (1.4)	19.1 (1.5)	17.4 (4.4)	18.1 (1.7)	12.1 (2.2)
**BMI, kg/m^2**	28.9 (0.1)	28.7 (0.1)	29.6 (0.3)	30.4 (0.3)	29.7 (0.6)	29.8 (0.2)	30.4 (0.6)
**CHD, %**	2.8 (0.2)	1.9 (0.1)	2.4 (0.5)	4.1 (0.5)	11.3 (3.4)	12.3 (1.5)	17.0 (2.9)
**Angina, %**	1.8 (0.1)	1.3 (0.1)	1.6 (0.4)	2.5 (0.4)	7.4 (2.9)	6.9 (1.1)	10.1 (2.7)
**MI, %**	2.7 (0.1)	1.9 (0.1)	2.1 (0.4)	4.0 (0.4)	8.4 (2.7)	12.0 (1.2)	17.9 (2.0)
**Stroke, %**	2.3 (0.1)	1.5 (0.1)	2.7 (0.5)	4.2 (0.6)	4.6 (1.2)	9.4 (0.9)	18.5 (2.1)
**CHF, %**	1.9 (0.1)	1.0 (0.1)	2.0 (0.4)	4.0 (0.5)	6.8 (1.8)	9.3 (1.0)	21.7 (2.6)
**COPD, %**	5.9 (0.3)	5.1 (0.3)	6.8 (0.8)	10.3 (1.0)	7.9 (1.7)	10.6 (1.1)	14.5 (2.5)
**Diabetes, %**	13.2 (0.4)	10.1 (0.3)	15.1 (1.2)	29.0 (1.8)	36.3 (3.3)	33.1 (1.8)	49.4 (3.9)
**Hypertension, %**	35.0 (0.5)	30.5 (0.6)	35.7 (1.8)	53.0 (1.6)	62.6 (3.5)	74.6 (1.7)	91.4 (1.3)
**Hyperlipidemia, %**	66.9 (0.5)	65.6 (0.5)	53.5 (1.7)	75.9 (1.5)	67.6 (4.0)	88.0 (1.1)	85.8 (2.0)
**Arthritis, %**	22.6 (0.5)	19.9 (0.5)	24.2 (1.6)	30.9 (1.6)	31.1 (3.5)	49.2 (2.3)	58.1 (3.1)
**Hemoglobin (g/dL)**	14.3 (0.0)	14.5 (0.0)	11.3 (0.0)	14.4 (0.0)	11.2 (0.1)	14.0 (0.0)	11.3 (0.1)
**eGFR (ml/min/1.73 m^2)**	96.1 (0.3)	98.8 (0.3)	102.5 (0.8)	97.3 (0.6)	95.9 (1.5)	50.1 (0.4)	41.5 (0.7)
**ACR**	28.3 (1.3)	7.6 (0.1)	8.8 (0.2)	166.0 (10.7)	240.1 (38.7)	125.3 (20.8)	322.8 (43.3)

Abbreviations: ACR, albumin-creatinine ratio; CHD, coronary heart disease; MI, myocardial infarction; CHF, chronic heart failure; CKD, chronic kidney disease COPD, chronic obstructive pulmonary disease; eGFR, estimated glomerular filtration rate.

Anemia status was classified using World Health Organization criteria (hemoglobin levels <12 g/dL in women and <13 g/dL in men); CKD was classified using KDOQI 2002 criteria (CKD Stage 1, albuminuria and eGFR ≥ 90 ml/min/1.73 m^2; CKD Stage 2, albuminuria and eGFR 60-89 ml/min/1.73 m^2; CKD Stage 3, eGFR 30-59 ml/min/1.73 m^2; CKD Stage 4, eGFR 15-29 ml/min/1.73 m^2; CKD Stage 5, eGFR < 15 ml/min/1.73 m^2.

Values in the Table are mean (SE) or percentage (SE).

Data were available on all sample participants (N = 22,933), except for education (data available in 22,915 participants), marital status (data available in 22,925 participants), house income (data available in 20,939 participants), health insurance (data available in 22,911 participants), employment (data available in 22,919 participants), smoking status (data available in 22,916 participants), alcohol use (data available in 17,827 participants).

Supplemental Table 3. Weekly MET-minute score by chronic kidney disease and anemia status, National Health and Nutrition Survey 2007–2016.

Overall	No CKD		CKD stages 1–2		CKD stages 3–5	
	No anemia	Anemia	No Anemia	Anemia	No anemia	Anemia
3428.9 (66.4)	3657.3 (76.2)	2596.8 (174.9)	2979.8 (168.7)	1943.9 (317.7)	1567.2 (129.0)	785.8 (85.2)

Abbreviations: CKD, chronic kidney disease; MET, metabolic equivalent.
Anemia status was classified using World Health Organization criteria (hemoglobin levels <12 g/dL in women and <13 g/dL in men); CKD was classified using KDOQI 2002 criteria (CKD Stage 1, albuminuria and eGFR ≥ 90 ml/min/1.73 m^2; CKD Stage 2, albuminuria and eGFR 60-89 ml/min/1.73 m^2; CKD Stage 3, eGFR 30-59 ml/min/1.73 m^2; CKD Stage 4, eGFR 15-29 ml/min/1.73 m^2; CKD Stage 5, eGFR < 15 ml/min/1.73 m^2.
Values in the Table are mean (standard error).

Supplemental Table 4. Percentage of participants with low (< 500 MET-minute per week), moderate (500 to 1000 MET-minute per week) and high (> 1000 MET-minute per week) physical activity by chronic kidney disease and anemia status, National Health and Nutrition Survey 2007–2016.

Overall	No CKD		CKD stages 1–2		CKD stages 3–5	
	No anemia	Anemia	No Anemia	Anemia	No anemia	Anemia
**Low (< 500 MET-minutes per week)**						
39.1 (0.6)	36.1 (0.6)	48.5 (1.9)	47.9 (1.9)	60.6 (3.5)	60.8 (2.3)	71.5 (2.9)
**Moderate (500 to 1000 MET-minutes per week)**						
9.5 (0.3)	9.8 (0.3)	8.7 (1.0)	8.0 (0.8)	8.3 (2.1)	7.4 (1.1)	9.6 (2.6)
**High (> 1000 MET-minutes per week)**						
51.4 (0.6)	54.1 (0.6)	42.8 (1.8)	44.1 (1.8)	31.1 (3.6)	31.8 (2.2)	18.9 (2.1)

Abbreviations: CKD, chronic kidney disease; MET, metabolic equivalent.
Anemia status was classified using World Health Organization criteria (hemoglobin levels <12 g/dL in women and <13 g/dL in men); CKD was classified using KDOQI 2002 criteria (CKD Stage 1, albuminuria and eGFR ≥ 90 ml/min/1.73 m^2; CKD Stage 2, albuminuria and eGFR 60-89 ml/min/1.73 m^2; CKD Stage 3, eGFR 30-59 ml/min/1.73 m^2; CKD Stage 4, eGFR 15-29 ml/min/1.73 m^2; CKD Stage 5, eGFR < 15 ml/min/1.73 m^2.
Values in the Table are percentage (standard error).

Supplemental Table 5. Adjusted mean differences (95% CI) in Weekly MET-minute score comparing participants with and without anemia by chronic kidney disease categories, National Health and Nutrition Survey 2007–2016.

	**No CKD**		**CKD stages 1–2**		**CKD stages 3–5**	
	**No anemia**	**Anemia**	**No Anemia**	**Anemia**	**No anemia**	**Anemia**
**Weekly MET-minute activity score**						
Model 1	Ref	-225.6 (-556.3, 105.1)	Ref	-596.2 (-1301.4, 109.0)	Ref	**-641.1 (-947.6, -334.6)**
Model 2	Ref	-75.8 (-411.7, 260.1)	Ref	-335.6 (-1056.9, 385.7)	Ref	**-517.4 (-819.9, -214.8)**
Model 3	Ref	68.7 (-269.9, 407.2)	Ref	-237.0 (-956.4, 482.4)	Ref	**-403.9 (-714.1, -93.7)**

Abbreviations: CKD, chronic kidney disease; MET, metabolic equivalent.
Anemia status was classified using World Health Organization criteria (hemoglobin levels <12 g/dL in women and <13 g/dL in men); CKD was classified using KDOQI 2002 criteria (CKD Stage 1, albuminuria and eGFR ≥ 90 ml/min/1.73 m^2; CKD Stage 2, albuminuria and eGFR 60-89 ml/min/1.73 m^2; CKD Stage 3, eGFR 30-59 ml/min/1.73 m^2; CKD Stage 4, eGFR 15-29 ml/min/1.73 m^2; CKD Stage 5, eGFR < 15 ml/min/1.73 m^2.
Model 1 adjusted for age, sex, race/ethnicity, and survey cycle.
Model 2 further adjusted for education, marital status, household income, health insurance, maintained employment, smoking status, alcohol use and comorbidities (congestive heart failure, coronary heart disease, angina pectoris, heart attack, stroke, arthritis, chronic obstructive pulmonary disease).
Model 3 further adjusted for serum iron, urine albumin to creatinine ratio, body mass index, hypertension, diabetes, and hyperlipidemia.
Numbers in bold type indicate interaction of CKD category by anemia group <0.05.

Supplemental Table 6. Adjusted prevalence rate ratios (95% CI) by Weekly MET-minute score category comparing participants with and without anemia by chronic kidney disease categories, National Health and Nutrition Survey 2007 – 2016.

Overall	No CKD		CKD stages 1–2		CKD stages 3–5	
	No anemia	Anemia	No Anemia	Anemia	No anemia	Anemia
**Low (< 500 MET-minute per week), base**						
**Moderate (500 to 1000 MET-minutes per week)**						
Model 1	Ref	0.75 (0.56, 1.00)	Ref	0.94 (0.51, 1.72)	Ref	1.21 (0.56, 2.65)
Model 2	Ref	**0.75 (0.56, 0.99)**	Ref	0.89 (0.49, 1.63)	Ref	1.39 (0.64, 2.99)
Model 3	Ref	0.81 (0.61, 1.07)	Ref	0.94 (0.51, 1.74)	Ref	1.44 (0.66, 3.15)
**High (> 1000 MET-minutes per week)**						
Model 1	Ref	**0.85 (0.72, 0.99)**	Ref	0.72 (0.51, 1.01)	Ref	**0.57 (0.42, 0.78)**
Model 2	Ref	0.88 (0.75, 1.04)	Ref	0.75 (0.53, 1.05)	Ref	**0.69 (0.50, 0.96)**
Model 3	Ref	0.94 (0.80, 1.10)	Ref	0.77 (0.55, 1.08)	Ref	0.74 (0.54, 1.01)

Abbreviations: CKD, chronic kidney disease; MET, metabolic equivalent.
Anemia status was classified using World Health Organization criteria (hemoglobin levels <12 g/dL in women and <13 g/dL in men); CKD was classified using KDOQI 2002 criteria (CKD Stage 1, albuminuria and eGFR ≥ 90 ml/min/1.73 m^2; CKD Stage 2, albuminuria and eGFR 60-89 ml/min/1.73 m^2; CKD Stage 3, eGFR 30-59 ml/min/1.73 m^2; CKD Stage 4, eGFR 15-29 ml/min/1.73 m^2; CKD Stage 5, eGFR < 15 ml/min/1.73 m^2.
Model 1 adjusted for age, sex, and race/ethnicity, and survey cycle.
Model 2 further adjusted for education, marital status, household income, health insurance, maintained employment, smoking status, alcohol use and comorbidities (congestive heart failure, coronary heart disease, angina pectoris, heart attack, stroke, arthritis, chronic obstructive pulmonary disease).
Model 3 further adjusted for serum iron, urine albumin to creatinine ratio, body mass index, hypertension, diabetes, and hyperlipidemia.
Numbers in bold type indicate interaction of CKD category by anemia group <0.05.

Supplemental Figure 1. Flowchart of study participants in the physical activity component, National Health and Nutrition Survey 2007–2016.

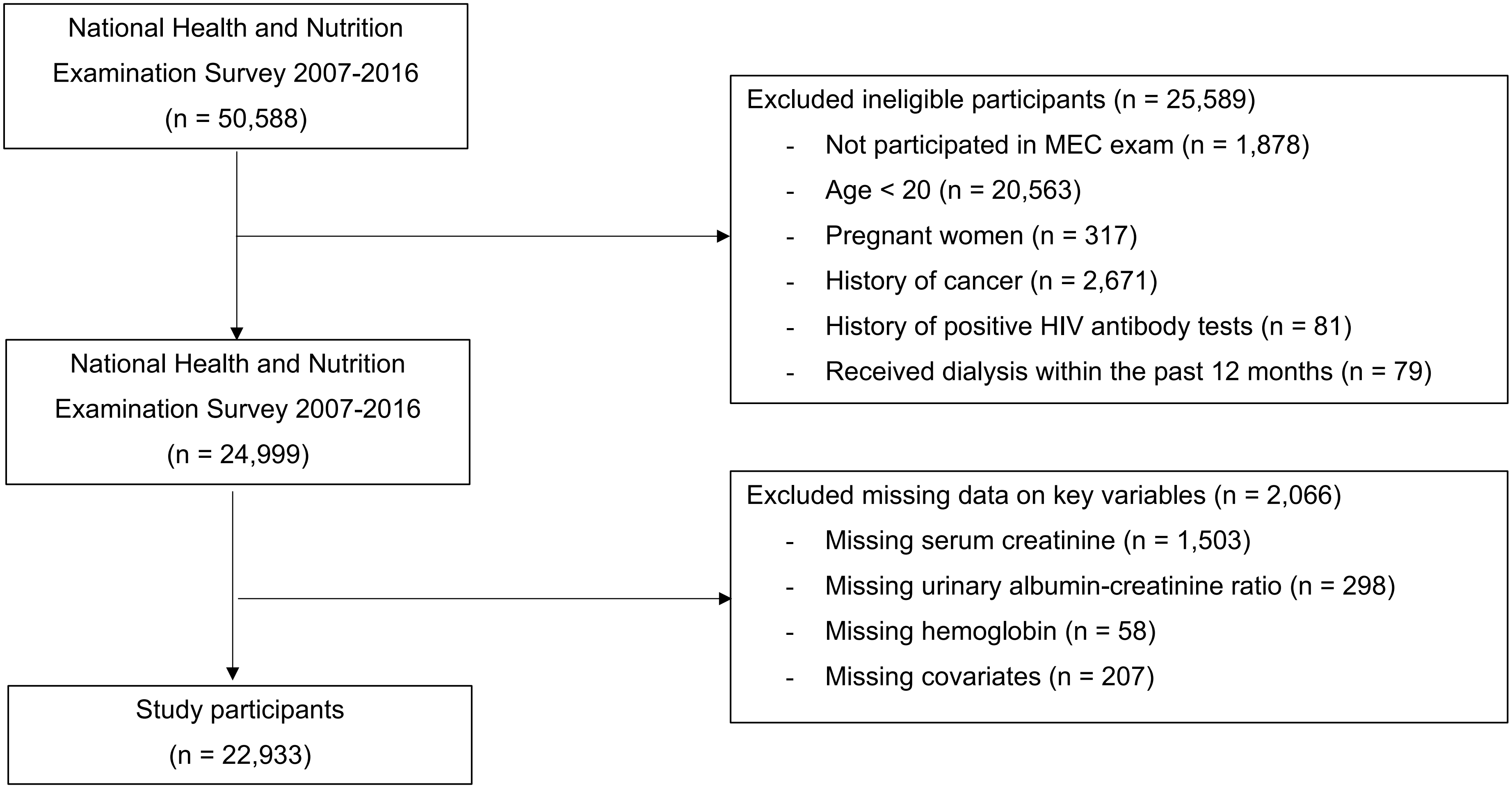

Supplement: Supplementary file 1 [file kidney360-4-e1212-s001.pdf]
